# Relationship between vitamin D status and caries experience in a group of Egyptian children: a cross-sectional study

**DOI:** 10.1186/s12903-023-03065-0

**Published:** 2023-06-09

**Authors:** Manal Ahmed El Shiekh, Rasha Mohamed Hatem Hanafy

**Affiliations:** Pediatric Dentistry & Dental Public Health, Faculty of Dentistry, Cairo University, Giza, Egypt

**Keywords:** Dental caries, Vitamin D, 25OHD (25 hydroxy vitamin D), Dmf, Oral hygiene

## Abstract

**Background:**

Dental caries is one of the most common diseases of childhood. Evidence suggests that malnutrition and vitamin deficiency may increase the risk to dental caries.

**Aim:**

This study aimed to determine the relationship between vitamin D and dental caries experience in children and whether vitamin D deficiency is a risk factor for tooth decay.

**Participants and methods:**

A cross-sectional study was performed on 51 Egyptian children, aged from three to five years and diagnosed from Abo El-Resh Children’s Hospital as ‘Sufficient’, ‘Insufficient’ or ‘Deficient’ in vitamin D. Children were divided into 3 equal groups. A structured questionnaire, formed of four sections, was answered by the parents. Dental examination was performed under natural daylight. Caries index (dmf), for each group, was calculated then compared. The study was conducted from July 2019 to January 2020. Associations between dmf and different variables were assessed using independent t-test. Correlation between age and dmf was assessed using Spearman’s rank order correlation coefficient. Multiple linear regression model was used to study the effect of different variables on caries.

**Results:**

There was a weak positive correlation between age and dmf scores (β = 2.00; 95%CI, 0.73:3.26). Children playing outside experienced higher dmf (β = 1.29; 95%CI, -0.35:2.94) than those with no outside play. Children with 25(OH) D below 20 ng / ml had the highest dmfs (β = 1.01; 95%CI, -0.74:2.76). There was a significant association with teeth brushing; children not brushing their teeth showed significantly higher dmf (β = -2.21; 95%CI, -4.14:-0.28) than their counterparts. There were no significant associations of sex (β = -1.05; 95%CI, -2.68:0.59), fluoride tablets intake (β = 2.19; 95%CI, -1.25:5.63), dental visits (β = -1.43; 95%CI, -3.09:0.23), mothers’ vitamin D intake during pregnancy (β = 0.71; 95%CI, -1.13:2.56), snacking (β = -1.18; 95%CI, -4.62:2.26) and parental education (β = 0.62; 95%CI, -1.18:2.42) with caries experience among the study population.

**Conclusion:**

Vitamin D deficiency does not seem to be associated with dental caries experience of 3–5 years old Egyptian children. Out of the indicator variables, age and tooth brushing contributed significantly to the occurrence of dental caries among the study population.

## Introduction

Dental caries is a very common disease of childhood that compromises the quality of life. It has a lot of complications that affect the nutritional health and growth of children including pain, high risk of recurrence, disturbed sleep, behavioral changes, and altered eating habits [1, 2]. Vitamin D is a fat-soluble vitamin, named calciferol, and has two forms: Vitamin D2, derived from plants, and vitamin D3, synthesized in human skin after exposure to ultraviolet B irradiation. Both forms of vitamin D remain metabolically inactive till their activation in the liver then in the kidney [3, 4].

Measuring plasma concentration of the circulating 25 Hydroxy vitamin D (25(OH) D) indicates the patient’s vitamin D status where 50–70 ng/ml is considered as optimal level [5, 6]. Prolonged decrease of vitamin D has detrimental effects including decreased mineralization of collagen matrix due to decreased serum calcium levels and increased parathyroid hormone, and increased risk for dental caries due to decreased mineralization of enamel and dentin resulting in enlarged pulp chambers and dentinal defects as in rickets [7–9]

Vitamin D deficiency in pregnancy, during periods of tooth development, also affects the tooth structure causing enamel hypoplasia and leading to increased caries risk [10, 11]. During childhood, vitamin D can help in preventing and treating dental caries by increasing calcium absorption leading to increased serum calcium levels, promoting mineralization of enamel and decreasing its demineralization [6].

Unfortunately, in Egypt, to the best of our knowledge, there is a knowledge gap in the literature regarding the evidence of a correlation between vitamin D Status and dental caries experience among Egyptian children from three to five years old. Thus, the current study was carried out to test the null hypothesis that there is no correlation between serum vitamin D levels and caries experience among a group of Egyptian children, aged from three to five years old.

## Participants & methods

### Study design

This study was an observational cross-sectional study, conducted in the Pediatric Dentistry and Dental Public Health Department, Faculty of Dentistry, Cairo University, for a period of six months, from July 2019 to January 2020.

### Sample size estimation

A power analysis was designed to have adequate power to apply a two-sided statistical test of the null hypothesis that there is no correlation between vitamin D status and dental caries experience in children. By adopting an alpha (α) level of 0.05 (5%), a beta (β) level of 0.20 (20%), that is, power = 80%, and an effect size of (0.44) calculated based on the results of the study of ***Schroth et al.*** [12]; the predicted sample size was found to be a total of (51) children (17 in each group based on laboratory investigation of serum vitamin D level). Sample size calculation was performed using G*power Program (University of Düsseldorf, Düsseldorf, Germany). and G*3 power3: flexible statistical power analysis program for the social, behavioral and biomedical sciences.

### Study population

A total of 51 cooperative Egyptian children, aged three to five years old, of both genders, diagnosed with ‘Sufficient’, ‘Insufficient’ and ‘Deficient’ serum vitamin D level, using radioimmunoassay technique, and referred from the Malnutrition Clinic, Abo El-Resh Children’s Hospital to seek dental care, were included in the study. On the other side, children with diabetes, renal disorders, bleeding disorders, and those who previously received vitamin D supply treatment were excluded.

According to ***Holick*** [13], children were classified into ‘Sufficient’ when serum vitamin D level is ranging between 30 and 100 ng / ml, ‘Insufficient’ when it is 20–30 ng / ml, and ‘Deficient’ less than 20 ng / ml.

### Informed consent

Prior to dental examination, a written consent from the parent/guardian was obtained after a comprehensive explanation of the study protocol.

### Data collection

The validated questionnaire of the previous study of ***Laksmiastuti et al., (2017)*** [14] was used with some modifications; repeated and incompatible questions to the outcome of this research were removed. For each child, the questionnaire, translated into Arabic, was answered by the parents. It was formed of four sections; Sect. 1, asked about child’s age, sex, address and medical history. Section 2, gathered data about dental care, dental habits, the use of supplements and dietary habits of the child. Section 3, assessed non- dental and dietary variables such as duration of exposure to sunlight. Section 4, gathered socio-demographic information as parents’ education level and occupation.

Dental examination was performed by a single well-trained calibrated investigator under natural daylight using a disposable diagnostic set (mirror, probe), while the child was seated on the dental chair, and caries index (dmf) was recorded according to the description of ***WHO,1997*** [15].

Before starting the study, the examiner was first trained to record (dmf) using photographs of carious teeth, with different degrees of severity, followed by live patients. Kappa value for intra-examiner calibration was 0.82.

### Addressing potential sources of bias

There was no risk of selection bias as all children attending the clinic the day of examination, and fulfilling the eligibility criteria were included in the study. Performance bias was avoided by using standardized methods by single well-trained calibrated investigator who recorded all data and performed the assessment. Reporting bias was avoided by reporting all data assessed.

### Statistical analysis

Categorical data were presented as frequencies and percentages. Numerical data were presented as mean and standard deviation values. They were checked for normality by checking the data distribution and by using Shapiro-Wilk’s test and were tested for variance homogeneity using Levene’s test. Data were normally distributed and the variance homogeneity assumption was not violated. Bivariate associations were tested using independent t-test. Correlation between age and dmf score was analyzed using Spearman’s rank order correlation coefficient since the linearity assumption was violated when the correlation was viewed on a scatter plot. Multiple linear regression model was built to assess the effect of different studied predictors on dmf score. Assumptions for the regression model were tested according to the methods devised by ***Fox***, (***2019)*** [16]. Linearity was assessed by partial regression plots and a plot of studentized residuals against the predicted values. Independence of residuals was assessed by a Durbin-Watson test. Homoscedasticity was assessed by visual inspection of a plot of studentized residuals versus unstandardized predicted values. Multicollinearity was assessed by inspection of VIF values. Outliers were assessed by checking Cook’s distance. Normality assumption was assessed by checking Q-Q plot. The significance level was set at p ≤ 0.05 within all tests. Statistical analysis was performed with R statistical analysis software version 4.2.3 for Windows ***(R Core Team, 2023)*** [17].

## Results

A total of 51 children were enrolled in this study to assess the relationship between serum vitamin D levels and caries experience in children. Group (1) included children diagnosed, as “Sufficient”, Group (2) “Insufficient”, and Group (3) “Deficient” in vitamin D levels. Among these children, 70.6% of the “Sufficient”, and 58.8% of the “Deficient” groups were males, while the majority of children in the “Insufficient” group (64.7%) were females. Furthermore, there was a significant difference between children ages in the three groups (***p*** = 0.002), where children of sufficient levels of vitamin D had a significantly lower mean age (3.67 ± 0.75) value than other groups (Table 1).

**Table 1:** Demographic data

Features				Vitamin D level			p-value
				Sufficient	Insufficient	Deficient	
**Gender**	**Male**		**n**	12	6	10	**0.145**
			**%**	70.6%	35.3%	58.8%	
	**Female**		**n**	5	11	7	
			**%**	29.4%	64.7%	41.2%	
**Age**		**Mean ± SD**		3.67 ± 0.75^B^	4.54 ± 0.57^ A^	4.25 ± 0.72^ A^	0.002*

The statistical analysis for gender and age was made using Chi square and One-way ANOVA tests respectively

Different superscript letters indicate a statistically significant difference within the same horizontal row*; significant (p ≤ 0.05)

Regarding the dmf scores in the three groups with different levels of vitamin D, there was a significant difference in dmf scores in the three groups (***p*** = 0.025). The highest mean dmf value was found in children with deficient levels (6.76 ± 2.68), followed by those with insufficient levels (5.71 ± 2.85), while the lowest value was found in children with sufficient levels of vitamin D (4.06 ± 2.54). Scores in children with deficient level were significantly higher than those with sufficient level (***p*** < 0.001).

Concerning the association and correlation with caries indicators, there was a weak positive correlation between age and dmf score that was statistically significant (rs = 0.327, p = 0.019). In addition, there was a significant association with outside play where children playing outside had significantly higher dmf scores (p = 0.024). There was a significant association with vitamin D level; where children with insufficient levels had significantly higher dmf score (p = 0.009). Moreover, there was a significant association with teeth brushing; children not brushing their teeth had significantly higher dmf score (p = 0.049). Other associations were not statistically significant (p > 0.05) (Table 2).

**Table 2:** Associations and correlations of dental caries experience with caries indicators among the study groups

Parameter		dmf (Mean ± SD)	Test statistic	p-value
**Sex**	***Male***	5.43 ± 2.82	-0.22	0.826
	***Female***	5.61 ± 2.98		
**Fluoride drops or tablets**	***No***	5.46 ± 2.95	-0.51	0.613
	***Yes***	6.33 ± 0.58		
**Dental visits**	***No***	5.59 ± 3.05	0.27	0.785
	***Yes***	5.35 ± 2.55		
**Outside play**	***No***	4.52 ± 2.31	-2.33	0.024*
	***Yes***	6.32 ± 3.06		
**Mother’s vitamin D intake during pregnancy**	***No***	5.39 ± 2.97	-0.60	0.552
	***Yes***	6.00 ± 2.49		
**Vitamin D level**	***Sufficient***	4.06 ± 2.54	-2.71	0.009*
	***Insufficient***	6.24 ± 2.77		
**Teeth brushing**	***No***	6.09 ± 2.80	2.02	0.049*
	***Yes***	4.44 ± 2.75		
**Snacking**	***No***	5.59 ± 3.05	-0.11	0.914
	***Yes***	5.35 ± 2.55		
**Parental educational level**	***Low***	5.62 ± 3.06	0.47	0.604
	***High***	5.17 ± 2.21		

Bivariate associations were tested using independent t-test. Correlation between age and dmf score was analyzed using Spearman’s rank order correlation coefficient

Correlation coefficient with age was 0.327 (95% CI, 0.056:0.552)

*; significant (p ≤ 0.05)

A multiple linear regression model was run to predict caries level (dmf score) from vitamin D as well as different confounders. There was linearity as assessed by partial regression plots and a plot of studentized residuals against the predicted values. There was independence of residuals, as assessed by a Durbin-Watson statistic of 1.91 (p = 0.646). There was homoscedasticity, as assessed by visual inspection of a plot of studentized residuals versus unstandardized predicted values. There was no evidence of multicollinearity, as assessed by tolerance values greater than 0.1. There were no studentized deleted residuals greater than ± 3 standard deviations, no leverage values greater than 0.2, and values for Cook’s distance above 1. The assumption of normality was met, as assessed by a Q-Q Plot.

The overall model was statistically significant (p = 0.007) and predicted 28.1% (adjusted R^2^) of the variability in dmf score. Out of the indicator variables, only age and tooth brushing were found to add significantly to the model (p < 0.05). It was found that age was significantly associated with higher dmf score (p = 0.003). In addition, teeth brushing was found to be significantly associated with lower dmf score (p = 0.026) (Table 3).

**Table 3:** Multiple linear regression model showing the relation between dental caries experience and socio-demographic, clinical and behavioral factors

*Parameter*	*Coefficient*	*Confidence level*		*SE*	*t-value*	*p-value*
		*Lower*	*Upper*			
*(Intercept)*	-1.75	-6.82	3.32	2.51	-0.7	**0.489**
*Age*	2.00	0.73	3.26	0.63	3.2	0.003*
*Sex (Female)* ^1^	-1.05	-2.68	0.59	0.81	-1.3	**0.202**
*Fluoride drops or tablets (Yes)* ^2^	2.19	-1.25	5.63	1.7	1.29	**0.205**
*Dental visit (Yes)* ^2^	-1.43	-3.09	0.23	0.82	-1.74	**0.089**
*Outside play (Yes)* ^2^	1.29	-0.35	2.94	0.81	1.59	**0.119**
*Mother’s vitamin D intake during pregnancy (Yes)* ^2^	0.71	-1.13	2.56	0.91	0.78	**0.440**
*Vitamin D (deficient)* ^3^	1.01	-0.74	2.76	0.87	1.17	**0.251**
*Brushing (Yes)* ^2^	-2.21	-4.14	-0.28	0.95	-2.32	0.026*
*Snacking (Yes)* ^2^	-1.18	-4.62	2.26	1.7	-0.69	**0.492**
*Parental educational level (High)* ^4^	0.62	-1.18	2.42	0.89	0.7	**0.491**

*; significant (p ≤ 0.05)

^1^ Reference category “Male”

^2^ Reference category “No”

^3^ Reference category “Sufficient”

^4^ Reference category “Low”

## Discussion

In the present study, the results of the multiple linear regression model used to study the effect of different variables on dental caries revealed no correlation between vitamin D status and dental caries experience, accepting the postulated null hypothesis.

In the current study, results revealed that highest dmf scores were present in children with “Deficient level” of vitamin D, while the lowest values were found in those with “Sufficient level”. This finding agrees with the results of other studies conducted by Williams et al. [18], in North America, Chen et al. [19], in Asia, and Schroth et al. [20], in Canada, and disagrees with Ahmed et al. [21], in Basra, who reported significantly higher 25(OH)D levels among caries-free preschool children.

Furthermore, children playing outside had significantly higher dmf scores, this could be attributed to the frequent purchase and intake of candies, chocolates and crackers from the accessible minimarkets while playing [22].

Surprisingly, the results of regression model performed to study the effect of different variables on dental caries revealed no correlation between serum vitamin D level and the occurrence of tooth decay and that vitamin D deficiency is not the main caries risk factor (p = 0.251). This may be due to limited sample size.

Furthermore, the variability in the dmf scores among the study groups is related, out of the indicator variables, to the “age” and “tooth brushing” (p < 0.05) rather than serum vitamin D deficiency. This goes in similarity with the finding of the study performed by Kim et al. [23], in Korea, which revealed no association between vitamin D levels and dental caries except for first molar caries occurrence, and Navarro et al. [24], in Netherlands, who suggested a weak association between serum vitamin D concentrations and risks of caries in primary teeth, and they didn’t recommend vitamin D supplementation for the prevention of dental caries in children.

Likewise, in this study, the regression model revealed that age was associated with higher dmf scores, this could be attributed to the fact that dental caries is a cumulative disease that increases by age [25]. Hence, primary prevention of dental caries is mandatory as soon as the primary teeth begin to erupt. This finding goes in accordance with those reported in Tanzania [26], Nigeria [27], and among Chinese children [28].

Moreover, in this study, tooth brushing was significantly associated with lower dmf scores proving that tooth brushing is an effective preventive measure of dental caries [29]. Also, tooth brushing is the most convenient way to remove dental plaque and deliver fluoride to the oral cavity, thus preventing dental caries [30–32].

### Clinical significance of the study

The results of this study revealed no correlation between vitamin D levels and dental caries experience. Among this study population, dental caries is mainly related to age and oral hygiene practice (tooth brushing) which may modify the belief of some children’s parents who always consider dental caries at the early age of their children to be related to vitamin D deficiency, subsequently, they abuse the use of multivitamins supplements. Moreover, it illustrates that primary prevention of dental caries as soon as the primary teeth begin to erupt is mandatory as dental caries is a cumulative disease that increases by age.

### Limitations of the study

The generalization of the results of this study is limited as it is a preliminary study. Furthermore, the actual nature of the relationship between vitamin D and dental caries is hard to define given the large number of variables that may contribute to both conditions. Also, the caregiver questionnaire involved retrospective questions, answered by the parents, which are subject to recall and response bias.

## Conclusion

Vitamin D deficiency does not seem to be associated with dental caries experience of 3–5 years old Egyptian children visiting a children’s hospital. Out of the indicator variables, only age and tooth brushing contributed significantly to the occurrence of dental caries among the study population.

## Data Availability

All data generated or analyzed during this study are included in this article.
